# Oncogene or Tumor Suppressor: The Coordinative Role of Lysine Methyltransferase SET7/9 in Cancer Development and the Related Mechanisms

**DOI:** 10.7150/jca.57663

**Published:** 2022-01-01

**Authors:** Ye Gu, Xiaofeng Zhang, Weiping Yu, Weifeng Dong

**Affiliations:** 1 Department of Gastroenterology, Key Laboratory of Clinical Cancer Pharmacology and Toxicology Research of Zhejiang Province, Affiliated Hangzhou First People's Hospital, Zhejiang University School of Medicine, Hangzhou, Zhejiang, P.R. China, 310006.; 2 Hangzhou Hospital & Institute of Digestive Diseases, Hangzhou, Zhejiang, P.R. China, 310006.; 3 Key Laboratory of Integrated Traditional Chinese and Western Medicine for Biliary and Pancreatic Diseases of Zhejiang Province, Hangzhou, Zhejiang, P.R. China, 310006.; 4 Department of Pathophysiology, Medical school of Southeast University, Nanjing, Jiangsu, P.R. China, 210009.; 5 Department of Laboratory Medicine, Cross Cancer Institute, University of Alberta, Edmonton, Alberta, Canada.

**Keywords:** SET7/9, lysine methyltransferase, methylation, cancer developments

## Abstract

SET7/9 is a member of the protein lysine methyltransferase family that methylates both histone 3 lysine 4 (H3-K4) and lysine(s) of other non-histone proteins. In recent years, dis-regulation of SET7/9 were frequently detected in various cancer types and SET7/9-mediated methylation has been recognized as an important mechanism that affects cancer initiation and development through regulation of a series of cellular processes. Here we review the currently identified histone and non-histone protein targets of SET7/9 that are closely correlated with human cancer and the function of SET7/9 in regulating the expression and stability of its protein targets. The review also discusses the putative role of SET7/9 as an oncogene or tumor suppressor in the development of various cancer types and the underlying mechanisms, which may help better evaluate the potential of SET7/9 as a novel candidate for cancer therapy.

## Introduction

Posttranslational modifications (PTMs) of histone proteins have a pivotal role in the dynamic transitions of chromatin structure in eukaryotes. Lysine methylation occurs on various histone lysine residues either site- or state-specifically, such as histone H3K4, H3K9, H3K79, and H4K20 1. It is a cardinal process underlying epigenetic modification of various tumor-related genes, leading to alterations in gene expression. So far many protein methylatransferases (PKMTs) that transfer different numbers of methyl groups from S-adenosyl-L-methionine (AdoMet) to the ε-amino group of the target lysine have been identified 2-5. Almost all of these protein methylatransferases harbor a conserved SET domain, which is initially identified in *Drosophila* 6-8. Recently, studies have revealed that PKMTs with conserved SET domain are not only responsible for histone lysine methylation, but also methylate various non-histone transcription factors important for a number of biological processes varying from cell growth, apoptosis, and stress response to DNA repair 9-13.

**Figure 1:**
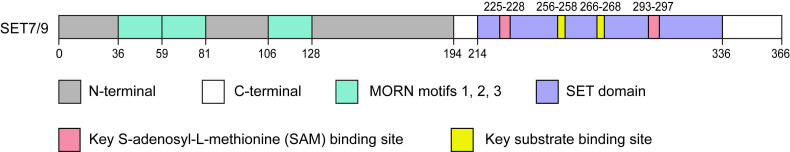
** Schematic diagram of SET7/9.** The SET7/9 protein contains an N-terminal segment and a C-terminal segment. The N-terminal helps stabilize SET7/9 protein and the C-terminal is mainly responsible for the catalytic function of SET7/9. The N-terminal contains three MORN motifs responsible for protein binding to plasma membrane phospholipids. The C-terminal contains the highly conserved SET domain. The cofactor SAM and protein substrate bind distinct sites on opposite surfaces of the SET domain.

SET7/9 ((Su(var)-3-9, Enhancer-of-Zeste, Trithorax) domain containing protein 7/9), also referred to as SET7, SET9, SETD7, or KMT7, is one of the best characterized members of the SET domain PKMTs family. *SET7/9* gene is located in human chromosome 4q28 with a protein product of approximately 50 kDa 14. Structural analyses have revealed that the SET7/9 protein is consists of an N-terminal segment and a C-terminal segment (Fig. 1). The N-terminal segment is non-conserved, while the C-terminal segment contains the highly conserved SET domain flanked by the pre-SET and post-SET region and inserted with an i-SET region 3,15,16. The cofactor and substrate bind in distinct clefts on the opposite surfaces of the SET domain 15-17 (Fig. 1). The N-terminal segment and pre-SET region in the C-terminal segment keep the structural stability of the protein 3,16. On the other hand, the residues in the i-SET and post-SET domains are involved in interactions with the substrate together with the core SET domain 16,17.

**Figure 2:**
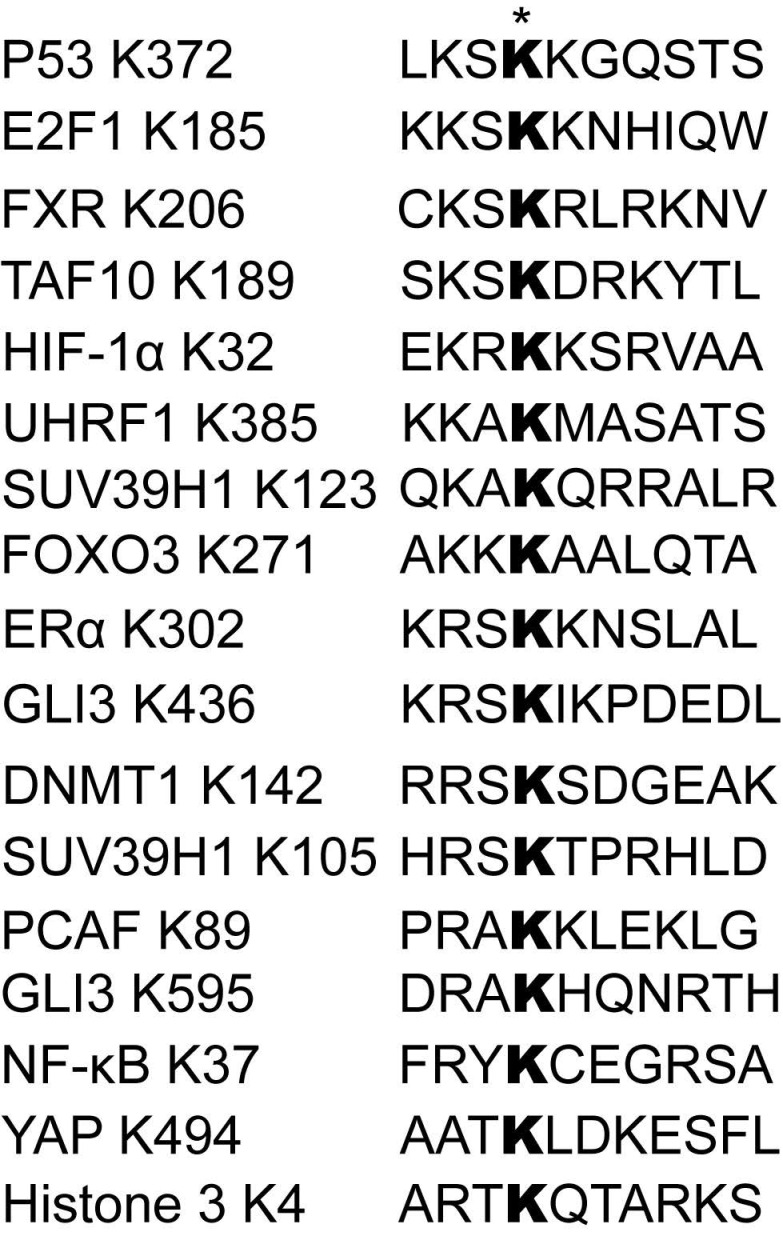
** Sequence alignment of a list of reported SET7/9 substrates and the recognition motifs.** The asterisk indicates the targeted lysine residues methylated by SET7/9.

SET7/9 was originally identified as a histone H3K4-specific methyltransferase 14. However, in the past decades many studies have found that SET7/9 preferentially targets non-histone proteins 18-23. Although generally considered as an enzyme catalyzing monomethylation, SET7/9 can also catalyze dimethylation of some substrates, such as the Msx2-interacting nuclear target protein (MINT) and ribosomal protein (Rpl29) 24,25. A consensus sequence motif of K/R-S/T/A-K with K being the target lysine residue was initially found to be required for successful recognition by SET7/9 26. However, a more recent peptide-array analysis has expanded the recognition motif to: (G/R/H/K/P/S/T)-(K>R)-(S>K/Y/A/R/T/P/N)-**K**-(Q/N>>other aa but not FYWPL)-(A/Q/G/M/S/P/T/Y/V), which implies a greater number of putative SET7/9 substrates to be discovered 24 (Fig. 2).

**Figure 3:**
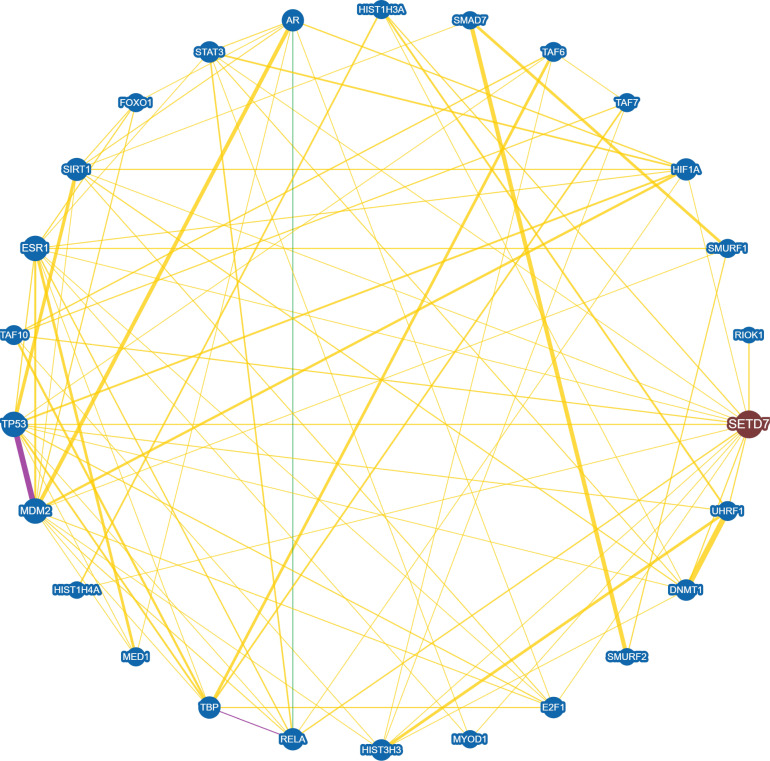
** Interaction network between SET7/9 and SET7/9 substrates with experimental evidences.** Protein interaction and genetic correlations are analyzed using the online tool BioGRID version 4.4 (https://thebiogrid.org/). Yellow line indicates direct physical interaction between two proteins. Green line indicates genetic interaction between two proteins. Purple line indicates both physical and genetic interaction between two proteins.

SET7/9-catalyzed histone H3K4 methylation and non-histone protein methylation often result in altered gene and protein expression 27,28. In an endothelial model of SET7/9 knockdown, more than 8,000 genes were transcriptionally dis-regulated and hundreds of loci in regulatory elements lost H3K4me1, as revealed by RNA-seq and ChIP-seq 28. The protein substrates of SET7/9 are often involved in the regulation of cellular processes including cell cycle, cell proliferation and differentiation, chromatin modulation, and DNA damage response. Meanwhile, many of these substrates also interact with each other, forming a complicated regulatory network (Fig. 3). Therefore, SET7/9-mediated methylation is an important mechanism that controls a series of physiological and pathological processes. Here we reviewed all the methylation targets of SET7/9 that are closely correlated with human cancer, and discussed the role of SET7/9 in cancer development through involvement in the regulation of cancer-related genes at both the transcriptional and posttranscriptional levels.

## Non-histone protein targets of SET7/9 and the biological functions

### p53

p53 is a transcription factor frequently mutated in human cancer 29. It acts as a tumor suppressor protein that induces cell apoptosis and cell cycle arrest upon DNA damage via activation of its downstream target genes 29,30. Upon DNA damage, p53 exerts its function by activating its downstream transcriptional targets, including pro-apoptotic factors Bax, Puma, Noxa, and p21/WAF/CIP. Protein p21 then inhibits the activities of cyclin-dependent kinases cdk2 and cdk1 (cdc2) as well as their partners cyclin E and cyclin B and promotes cell cycle arrest 31.

**Figure 4:**
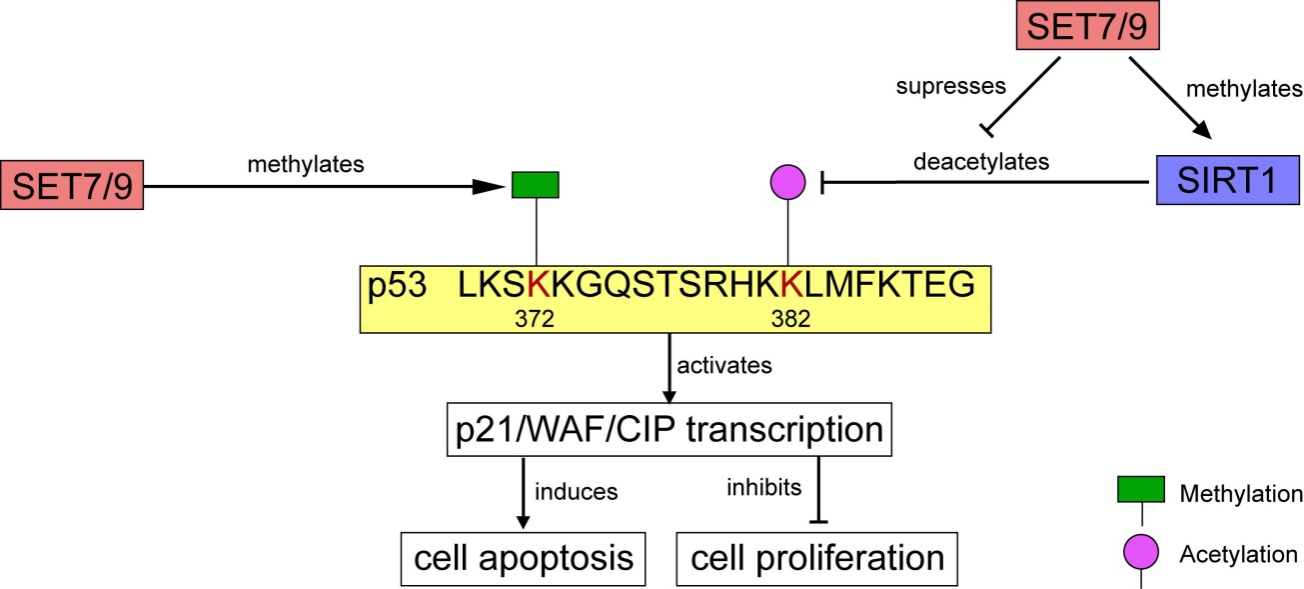
** Regulation of p53 activity by SET7/9.** On one hand, SET7/9-mediated methylation at p53 K372 stimulates subsequent acetylation and stabilization of p53. On the other hand, SET7/9 suppresses the interaction of SIRT1 and p53, thus abrogating SIRT1-mediated deacetylation of p53. The stabilized p53 protein further activates transcription of *p21/WAF/CIP*, induces cell apoptosis and inhibits cell proliferation.

**Figure 5:**
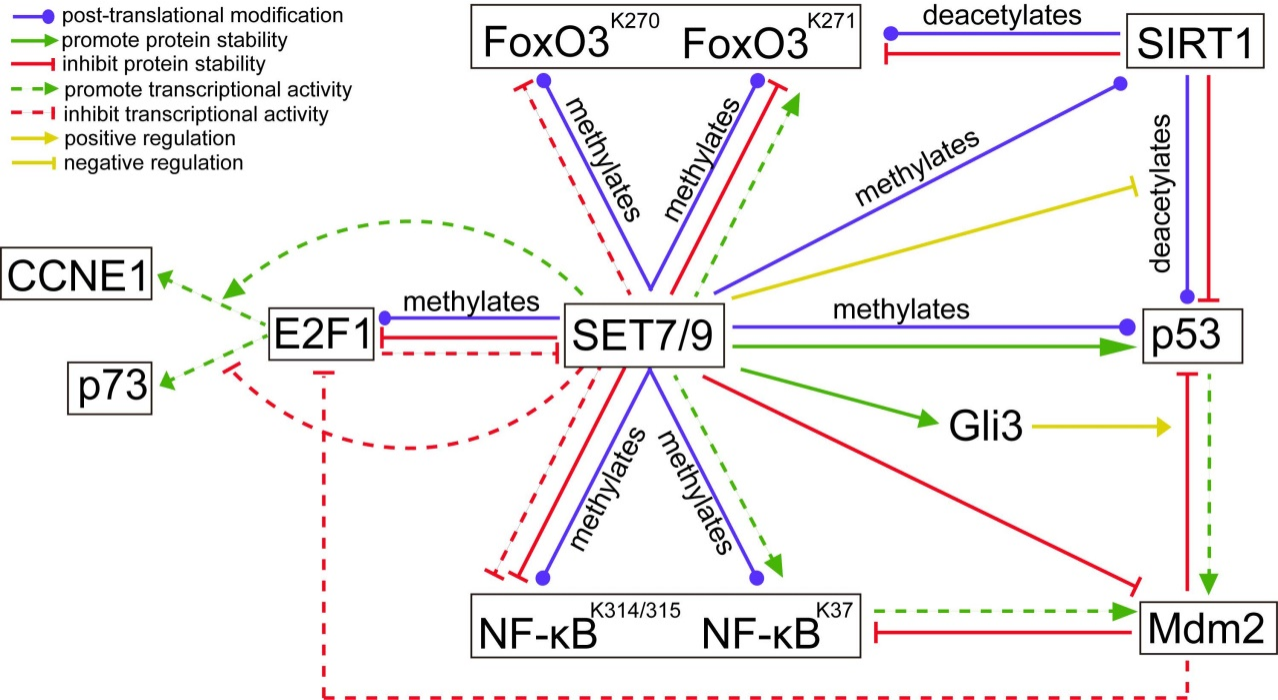
** The regulatory network between known SET7/9 substrates that are important transcriptional factors involved in cancer development.** The action type and effects depict the type of regulation between two proteins.

In human osteosarcoma cells U2OS, p21/WAF/CIP was modulated through the SET7/9-p53 axis in response to DNA damage 18. SET7/9 catalyzes mono-methylation of p53 at K372. The methylation level is enhanced by DNA damage, which stimulates subsequent acetylation of p53 and contributes to the stabilization and retention of p53 in the nucleus 9,18 (Figs. 4, 5). Ultimately, the p21/WAF/CIP gene is activated and cell cycle arrest is induced 18 (Fig. 4). SET7/9-silenced U2OS cells were unable to undergo G2/M arrest, and expression of both p53 and p21 were remarkably decreased 18. Furthermore, in colorectal cancer (CRC) cells HCT116, CO115, and SW480, function of SET7/9 is indispensable for resveratrol-driven p53 activation and cell apoptosis. Ablation of SET7/9 abolished resveratrol-induced overexpression of p53 as well as its downstream apoptotic markers PARP, and cleaved caspase-3 32. However, whether SET7/9 can directly regulate the expression of these pro-apoptotic factors or it only operates through p53 to activate them remained unknown.

Although results from *in-vitro* studies have suggested an anti-tumor effect of SET7/9 through positive regulation of p53, the indispensible role of SET7/9 in the stabilization of p53 and regulation of p53-mediated DNA damage response is questioned by vivo experiments. Lehnertz et al. (2011) investigated the involvement of SET7/9 in p53 regulation using an independent knockout mouse strain with SET7/9 deficiency 33. However, no defect in p53-dependent transcription following genotoxic and oncogenic insults was detected in cells from these mice. The canonical p53 functions were not impaired, either 33. The results were confirmed by the study of Campaner et al. (2011), which further showed that SET7/9 was dispensable for p53 acetylation as well as p53-dependent cell-cycle arrest or apoptosis in mouse 34. Unlike p53-deficiency, SET7/9-deficiency did not accelerate tumor onset and affect mouse survival and physiology in a murine model of Myc-induced lymphoma 33. The most reasonable explanation for the disparity of *in-vitro* and *in-vivo* studies is that methylation of p53 by SET7/9 has no functional effect on p53 function. In support of this, no effect on the stabilization and activation of p53 was observed after substitution of the SET7/9 methylation site with arginine in mouse embryo fibroblasts (MEFs) 35. Considering the complexity of cancer initiation and progression, which may result from dis-regulation of various oncogenes and tumor suppressor genes, abnormal SET7/9 expression might become a carcinogenic factor only when all other p53 regulatory pathways are disabled 33.

### E2F1

E2F1 belongs to the E2F transcription factor family. It is a key regulator of various cellular stress- and apoptosis-related genes 36. By activating its downstream target gene *p73*, E2F1 can induce cell apoptosis independent of p53 37. Meanwhile, it also regulates the expression of *CCNE*, which encodes a protein named cyclin E that can directly interact with cdk2 to accelerate DNA replication and cell proliferation 38.

Unlike the stabilization effect brought about by SET7/9-mediated methylation on p53, SET7/9-mediated methylation of E2F1 K185 interferes with acetylation and phosphorylation of the protein, thus promotes its ubiquitylation and subsequent degradation 39,40 (Fig. 5). Hence, SET7/9 can negatively influence E2F1-mediated cell death independent of the p53-axis 19,39,41. In p53-deficient lung cancer cells H1299, overexpression of SET7/9 de-stabilized E2F1 protein and prevented activation of *p73*, a downstream pro-apoptotic target of E2F1, thus inhibited cell apoptosis via a p53-independent pathway 39,41. In turn, E2F1 may repress SET7/9 transcription in cooperation with Snail 40. In breast cancer cells MCF7, silencing E2F1 resulted in significantly decreased Snail occupancy on both SET7/9 and E-cadherin promoters and evoked EMT transition 40 (Fig. 5).

In addition to direct methylation of E2F1, SET7/9 also modulates the binding of E2F1 on the promoter of its target genes. Chromatin immunoprecipitation (ChIP) assay revealed that SET7/9 co-activated *CCNE1* transcription but repressed *p73* transcription downstream of E2F1 through chromatin modification 19 (Fig. 5). As a result, increased number of SET7/9-silenced cells in G1/S after genotoxic stress was observed in both H1299 and U2OS cell line irrespectively of the p53 status and source of DNA damage 19. This suggests that SET7/9 is located upstream of E2F1 in the signal transduction cascade of DNA damage response, coordinating the final transcriptional outcome.

On the contrary, in hepatocellular carcinoma (HCC) cells Huh7 and colorectal cancer cells HCT116, SET7/9 stabilized E2F1 and up-regulated E2F1 downstream targets 42,43. Decreased cell proliferation, migration and invasion were observed in SET7/9-silenced Huh7 cells as well as in wild‑type Huh7 cells treated with MTA, suggesting that SET7/9 may be responsible for HCC development through post‑translational regulation of E2F1 43. It was postulated that SET7/9-catalyzed methylation may lead to discrepant effects in the stability of its target protein depending on the types and amounts of post-translational modifiers under different cell contexts 43.

### Mdm2

Except for the p53- and E2F1-dependent regulation of DNA damage response, SET7/9 can also affect DNA damage response and DNA repair via Mdm2, an E3 ubiquitin ligase that regulates the both p53 and E2F1 44. Previous studies have shown that Mdm2 blunts the transcriptional activity of E2F1 without triggering its degradation 45 (Fig. 5). Meanwhile, it inactivates p53 by targeting p53 for ubiquitin-mediated proteasomal degradation and interacting with and obscuring the N-terminal trans-activation domain of p53 46. In turn, p53 transcriptionally activates expression of Mdm2 46 (Fig. 5). For cancers with wild-type or functional p53, targeting the interaction between MDM2 and p53 represents an attractive treatment approach 47. Similarly, Mdm2 can bind to and inhibit the p65/RelA subunit of NF-κB, which also transcriptionally activates Mdm2 expression 48 (Fig. 5). The involvement of Mdm2 in the negative feedback loops with p53 and NF-κB has provided a novel mechanism of crosstalk between the p53 and NF-κB pathways.

Although direct methylation of Mdm2 by SET7/9 was not detected, evidences from GST pull-down assay proved that SET7/9 can physically interact with Mdm2, binding to its amino- and carboxyl-termini and decreased Mdm2 expression 44 (Fig. 5). In SET7/9-silenced U2OS cells, expression of p53 was down-regulated while expression of Mdm2 was up-regulated upon genotoxic stress 44. Attenuation of SET7/9 led to increased DNA damage sensitivity and impaired DNA repair, while knockdown of both SET7/9 and Mdm2 negated the effect caused by knockdown of SET7/9 alone. The study implicated that SET7/9 operates in DNA damage response, at least partially via Mdm2 by acting as an antagonistic regulator. However, the exact consequences of interaction between SET7/9 and Mdm2 have remained unclear. As expression of Mdm2 also depends on transcriptional factors such as p53 or p73, it is unknown whether SET7/9 directly methylates Mdm2 and affects Mdm2 stabilization or indirectly suppresses Mdm2 expression via other regulators of Mdm2. Considering the physical binding of Mdm2 with several chromatin-associated factors, including E2F1, Tip60 and NBS1 49, downstream effectors of the SET7/9-Mdm2 axis and related pathways should also been investigated to improve our understanding of SET7/9 in the molecular mechanisms of DNA damage in cancer cells.

### SIRT1

Apart from transcriptional factors, the methylation substrates of SET7/9 also include many histone modifiers, such as Sirtuin 1 (SIRT1), a mammalian nicotinamide adenine dinucleotide (NAD^+^)-dependentclass III histone deacetylase (HDAC) 50. SIRT1 is generally considered as an oncogenic protein, which catalyzes deacetylation of several known tumor suppressors including p53 and FoxO 51,52. Elevated SIRT1 expression is detected in various cancer tissues and cancer cell lines, such as leukemia and prostate cancer 53,54. In HEK293T cells and HCT116 cells, SET7/9 directly interacts with SIRT1 and the interaction was markedly enhanced in response to DNA damage 50. The interaction between SET7/9 and SIRT1 facilitated the dissociation of p53 from SIRT1, stabilized the acetylation level of p53, and increased p53-mediated transactivation 50 (Figs. 4, 5). In vitro methylation assay and mass spectrometry confirmed that SET7/9 catalyzes SIRT1 methylation at K233, K235, K236, and K238 50,55. Nevertheless, these methylation events do not affect the deacetylase activity of SIRT1. To explain the dissociation of p53 from SIRT1 mediated by SET7/9 and SIRT1 interaction, the authors proposed that SET7/9 may compete with p53 for binding to SIRT1, or methylation of SIRT1 by SET7/9 may induce conformational change in SIRT1 that prevents SIRT1 from binding to p53 50. Testing of these two hypotheses may provide a more complete understanding of how SIRT1 is regulated and how it in turn regulates its downstream targets, which will be valuable in the design of new anti-cancer therapies 50.

### NF-κB

NF-κB is an important activator of cellular inflammatory responses with pathological implications. NF-κB mainly resides in the cytoplasm in an active form in normal un-stimulated cells. Upon multiple extracellular stimuli such as TNF-α-treatment, activated NF-κB translocates into the nucleus and binds to the promoters of a diverse array of genes, through which NF-κB may promote the growth and survival of many solid and haematological maligancies 56. NF-κB consists of homo- or heterodimers composed of many different subunits, with p65/RelA and p50 being the best characterized 57.

Using mass spectrometric analysis, the p65/RelA subunit was found to be methylated at K314 or/and K315 by SET7/9 13. Methylation of p65/RelA induces its ubiquitination and proteasome-mediated degradation and represses the transcription activity of NF-κB 13 (Fig. 5). In U2OS and A549 cells, knockdown of SET7/9 or mutation at K314 or K315 prolonged DNA binding of NF-κB to its target genes in response to TNF-α, indicating SET7/9-mediated negative regulation of NF-κB activation 13. Consistently, a study using human U266 multiple myeloma cell line showed that berberine treatment increased SET7/9 expression, promoted p65/RelA methylation, and suppressed p65/RelA-dependent transactivation of miR-21. Berberine induced cell apoptosis and inhibited cell proliferation in a dose-dependent manner, while knockdown of SET7/9 increased the nuclear level of p65/RelA and partially recovered cell proliferation 58.

Dramatically, SET7/9-mediated mono-methylation of a different lysine site, K37 of NF-κB may lead to the completely opposite effects. In HeLa cells, methylation of NF-κB prompted interaction between p65/RelA and its target sequences, and selectively activating the expression of a subset of genes downstream of NF-κB 20 (Fig. 5). A possible explanation for the different regulatory effects of SET7/9 on NF-κB activity is that in HeLa cells p65/RelA K37 is exclusively methylated by SET7/9, while in U2OS cells p65/RelA K37 can be modified by other methyltransferases 20. Similarly, silencing of SET7/9 in airway smooth muscle (ASM) cells decreased activation of NF-κB and inhibited TNF-α-induced cell proliferation and migration, but whether the regulatory effect is related with protein methylation was not reported 59.

### FoxO3

The Forkhead Box O (FoxO) transcription factors are potent transcriptional activators regulating the expression of a wide range of target genes involved in stress response, cell metabolism, differentiation, and cell apoptosis. In mammal cells, FoxO transcriptional factors play a conserved role in tumor suppression. Activation of the FoxO member FoxO3 induces cell cycle arrest and promotes cell apoptosis in gastric cancer, pancreatic cancer, cervical cancer 60,61, while loss of FoxO3 is associated with poor prognosis in estrogen-dependent breast cancer 62. With the increased understanding of FoxO3, it is also found to be responsible for the sensitization of cancer cells to chemotherapy, which is of great clinical significance 63.

As the other members of the FoxO family, FoxO3 subjects to a post-translational modification at multiple lysine residues by a number of acetylases, methyltransferase, and ubiquitin enzymes. FoxO3 K270 and K271 were found to be two methylation targets of SET7/9 in 293T cells 64,65 (Fig. 5). SET7/9-mediated methylation of FoxO3 K270 inhibited binding of FoxO3 to its target genes and suppressed FoxO3 transactivation activity, thus reduced oxidative stress-induced expression of Bim downstream of FoxO3 64. On the contrary, SET7/9-mediated methylation of FoxO3 K271, which is the site shown to be deacetylated by SIRT1, slightly increased FoxO3 transcriptional activity, but decreased FoxO3 stability 65 (Fig. 5). These results are not surprising given that SET7/9 can also inversely regulate the transcription of different downstream targets of E2F1 19. However, the biological effects of FoxO3 K270 and K271 methylation by SET7/9 need to be investigated in order to unravel the role of SET7/9-FoxO3 axis in cancer development. Meanwhile, whether the effects on FoxO3 activity and cellular biological feature caused by SET7/9 depend on the cell type or the type of target genes is also an interesting question to be addressed 65.

### Gli1/3

The Gli zinc finger transcription factors are important modulators of oncogenic Hedgehog signaling 66. Among the three Gli protein members, Gli1, Gli2, and Gli3, Gli3 has attracted great attention for its critical role in modulating the switch-on and -off of Shh signaling. Upon activation of Shh signaling, Gli3 transitions from a transcriptional repressor to a trans-activator that increases the expression of downstream targets including Gli1 and Ptch1 67,68. In pancreatic cancer, Gli3 mediates cell survival and increased cell resistance to cyclopamine, while in colorectal cancer, Gli3 augments tumorigenicity by up-regulating adherence-related genes 69,70.

The study of Fu et al. (2016) reported that SET7/9 specifically methylates the full-length Gli3 at K436 and K595, increasing the stability and DNA-binding capacity of Gli3 on the promoter of Gli1 71. Gli3 acts as a critical transcriptional activator and amplifier of Shh signal. As a result, methylation of Gli3 by SET7/9 led to sustained activation of Shh signaling, followed by accelerated tumor growth and metastasis of non-small cell lung cancer (NSCLC) 71. Moreover, the expression levels of SET7/9 and Gli1 showed a positive relationship in NSCLC tumor samples, indicating a regulatory role of the SET7/9-Gli3-Gli1 axis in NSCLC development 71. Interestingly, in colorectal cancer cells, loss of Gli3 led to stabilization and activation of p53 via inhibition of Mdm2-mediated p53 ubiquitination and degradation 72 (Fig. 5). Since SET7/9 is proved to be the direct regulator of Gli3, p53, and Mdm2, discovery of the regulatory network composing of SET7/9, Gli3, p53, and Mdm2 has reinforced the significance of SET7/9 in regulating carcinogenesis through multiple molecules.

### ERα and AR

Estrogen receptor α (ERα) and androgen receptor (AR) are nuclear hormone receptors essential for the regulation of cell differentiation, proliferation, and cell survival in breast cancer and prostate cancer. Once binding to estrogen or androgen, ERα and AR are translocated into the nucleus where they recruit co-activator complexes with histone acetyltransferase or methyltransferase activities to activate downstream target genes 12,73. Methylation of ER at K302 by SET7/9 prevents ER ubiquitylation and degradation and stabilizes the protein 12. Knockdown of SET7/9 resulted in a concomitant decrease in ERα expression and attenuated estrogen-driven transcriptional response. Meanwhile, binding of ER to its target genes such as *PS2* and progesterone receptor (*PgR*) was also inhibited after SET7/9 knockdown 12. In vitro and in vivo analyses also revealed an inner relationship between two amino acid sites of ER, that is, K302 methylation of ERα is suppressed by mutation at K303R, which has been linked to more aggressive clinical features of breast cancer 12,74,75. In order to determine the significance of K302 methylation in breast cancer development and evaluate the potential of SET7/9 as a biomarker or therapeutic target, the mutual impact between K302 methylation and other modifications of ERα needs to be investigated 12. Similarly, methylation of AR K630 by SET7/9 potentiates the transcriptional activity of AR 28,76. Although the effects of SET7/9-mediated ERα and AR methylation in cellular behavior were not examined, the importance of ERα and AR in stimulating cell proliferation and anti-apoptotic responses has implicated an indispensable role of SET7/9 in positive regulation of breast cancer and prostate cancer 77,78.

### YAP

**Figure 6:**
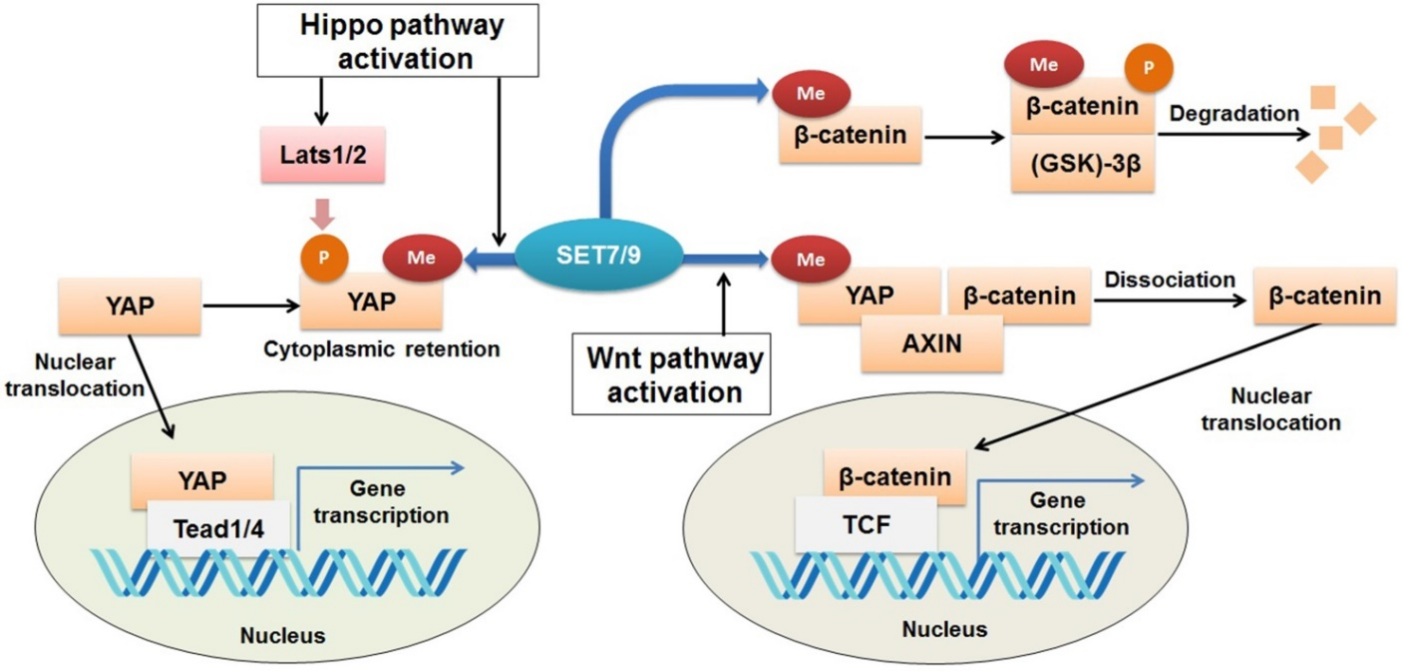
** Regulation of Wnt/β-catenin and Hippo signaling pathways by SET7/9.** SET7/9 can methylate YAP K494 and β-catenin K180. Under normal conditions, methylation of β-catenin by SET7/9 promotes GSK-3β-mediated ubiquitination and degradation of the protein. Upon Hippo signaling, methylation of YAP K494 by SET7/9 binds YAP in the cytoplasm, while upon Wnt signaling activation, methylation of YAP K494 by SET7/9 leads to dissociation of the YAP-AXIN-β-catenin complex, releases β-catenin and promotes its nuclear translocation, thus facilitates Wnt/β-catenin-dependent tumorigenesis.

The Yes-associated protein (YAP) functions as a transcriptional co-activator of genes controlling cell proliferation and apoptosis 79. It is a transducer of the Hippo signaling, which regulates organ size and function 80,81. Activation of Hippo signaling results from cell-cell contact, cell polarity, and other mechanical cues can ultimately lead to cytosolic retention and/or degradation of YAP (82). SET7/9 is required for the Hippo-mediated sequestration of YAP in the cytoplasm by methylation of K494 residue of the YAP protein 23 (Fig. 6). Lack of SET7/9 function in mice and murine embryonic fibroblasts (MEFs) resulted in accumulation of YAP in the nuclear and up-regulation of YAP-targeted genes 23,83. What's more, a larger progenitor compartment in the intestine in SET7/9-deficient mice was observed 23. Since dis-regulation of YAP is often associated with initiation of breast cancer, liver cancer, and colon cancer 84-86, the SET7/9-dependent dynamic regulation of YAP localization may be another possible mechanism through which SET7/9 can affect cancer progression.

### β-catenin

β-catenin is known to be an onco-protein and a key coordinator of the Wnt/β-catenin signaling pathway 87,88. Using mutagenesis assay and mass spectrometric analyses, a recent study has revealed that SET7/9 directly catalyzes mono-methylation of β-catenin K180 89 (Fig. 6). After methylation, β-catenin was subject to ubiquitination and subsequent degradation mediated by phosphokinase glycogen synthase kinase (GSK)-3β 89 (Fig. 6). In HeLa cells with silenced SET7/9 expression, transcription of the typical β-catenin downstream gene targets, *c-myc* and *cyclin D1*, was significantly up-regulated, indicating the function of SET7/9 in inhibiting cell growth and proliferation via methylation of β-catenin K180 89.

However, SET7/9 may cause the opposite consequences on the Wnt/β-catenin pathway, as reported by another study 90. In an established mouse intestine model, SET7/9 was responsible for higher susceptibility to tumorigenesis in the context of dis-regulated Wnt signaling. Wnt-dependent expression of the tumor stem cell marker *Lgr5* and the proto-oncogene *Myc* were reduced after SET7/9 knockdown, supporting the finding that SET7/9 facilitates Wnt/β-catenin-dependent tumorigenesis and regeneration 90. The Yes-associated protein (YAP) is involved in this regulatory network by constituting a complex with SET7/9 and β-catenin (Fig. 6). SET7/9-dependent methylation of YAP prompts translocation of β-catenin to the nuclear 23,90 (Fig. 6). The study has implicated the value of SET7/9 in clinical diagnosis and treatment for intestinal cancer 90. In addition, the regulation of the Wnt/β-catenin and Hippo/YAP pathways by SET7/9 revealed a methyltransferase-dependent mechanism that underlies the crosstalk between different signaling pathways during intestinal regeneration and tumorigenesis.

### HIF-1α

Hypoxia-inducible factor-1α (HIF-1α), a heterodimeric complex comprising of an oxygen-regulated α-subunit, is a hypoxia-inducible factor encoded by the HIFA gene. HIF-1α is a core transcription factor regulating cellular response to hypoxia signaling, which contributes to the aggressive through promoting cells to undergo the fundamental metabolism adaptation 91. Dis-regulated HIF-1α is often associated with altered expression of genes controlling angiogenesis, cell survival, and tumor invasion 92. In liquid chromatography mass spectrometry combined with co-immunoprecipitation analysis confirmed methylation of HIF-1α K32 by SET7/9, which leads to degradation of the protein by 26S proteasomes 93. In mouse embryonic fibroblasts (MEFs) under hypoxic stress, overexpression of SET7/9 led to reduced cell motility, while overexpression of lysine specific demethylase1 (LSD1) reversed the effect caused by SET7/9 and led to increased cell motility 93. Moreover, mice with mutated HIF-1α displaying methylation defects showed accelerated retinal tumor vascularization and tumor angiogenesis 93, 52. The results implicated a different pathway through which SET7/9 functions to affect human cancer development.

### pRb

**Figure 7:**
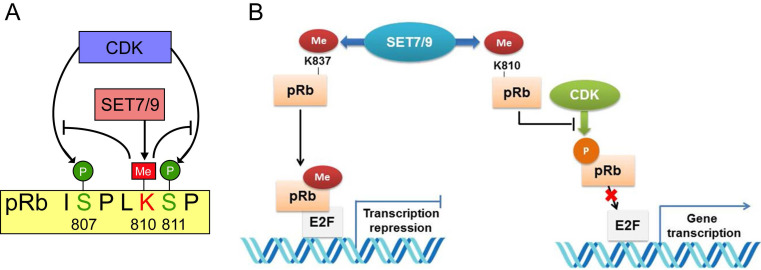
** Interplay between pRb lysine methylation by SET7/9 and phosphorylation by CDK in the control of cell cycle and cell growth. (A)** SET7/9 methylates pRb at K810 and hinders phosphorylation of pRb at S807 and S811 by CDK. **(B)** Methylation of pRb K837 is required for the repression of the E2F family transcription factors and pRb-dependent cell cycle arrest. Methylation of pRb K810 impedes phosphorylation of pRb and prevents dissociation of pRb from E2F transcription factors.

The retinoblastoma tumor suppressor protein (pRb) functions as a hub in the regulatory network orchestrating cell proliferation, differentiation, and survival 94. Inactivation of pRb not only allows for inappropriate cell proliferation, but also undermines mitotic fidelity, genome instability and ploidy changes, which further promote tumor growth, tumor relapse and resistance to therapeutics 95. Two lysine sites, K873 and K810 of pRb were found to be directly monomethylated by SET7/9 96-98. Methylation of pRb K873 is required for pRb-dependent cell cycle arrest and repression of the E2F family transcription factors 96 (Fig. 6). In osteosarcoma cell lines U2OS and SAOS2, knockdown of SET7/9 led to reduced cell cycle arrest and accumulation of protein products of many E2F target genes 96. Methylation of pRb K810, the core subunit facilitating cdk phosphorylation, hinders interaction between pRb and Cdk, thus inhibits pRb phosphorylation and augments cell growth inhibition 97 (Fig. 7). Methylated pRb K810 can also be recognized by p53 binding protein 1 (53BP1), forming a chromatin-bound pRb/53BP1 complex on E2F target genes. The interaction between 53BP1 and methylated pRb enables pRb to participate in pathways regulating cell cycle progression and influencing the DNA-damage response 98.

### UHRF1

Ubiquitin-like containing PHD Ring Finger 1 (UHRF1) is a key epigenetic regulator required for maintenance of DNA methylation and heterochromatin formation. UHRF1 is primarily expressed in proliferating cells, promoting S-phase entry. Up-regulation of UHRF1 may serve as a biomarker for a variety of cancers; including breast, gastric, prostate, lung and colorectal carcinoma 99-104. In HCT116 and H1299 cells, UHRF1 is methylated by SET7/9 at K385 in response to DNA damage 105. SET7/9-mediated methylation of UHRF1 promotes its interaction with proliferating cell nuclear antigen (PCNA), which functions in DNA replication and cell cycle regulation 105. Enhanced UHRF1-PCNA interaction further leads to polyubiquitination of PCNA and induction of homologous recombination and finally facilitates cell survival 105. HCT116 cells overexpressing UHRF1 showed increased cell viability whereas methylation-deficient mutants showed reduced cell viability 105. On the contrary, the histone H3K4 demethylase LSD1 can de-methylate UHRF1 and blocks polyubiquitination of PCNA 105. The methylation status of UHRF1 dynamically controlled by SET7/9 and LSD1 in double-strand break repair pathway is essential for cell viability and survival, which may contribute to tumor progression and metastasis 105.

### SUV39H1

Suppressor of variegation 3-9 homolog 1 (SUV39H1) is a histone methyltransferase that catalyzes H3K9 tri-methylation 106. The function of SUV39H1 in cancer development is pleiotropic. Generally, SUV39H1 is regarded as a tumor suppressor for its role in inhibition of genes required for cell proliferation 107. However, an oncogenic role of SUV39H1 has been reported in retioblastoma and clear cell renal cell carcinoma and up-regulation of SUV39H1 has been observed in several human cancers, such as colorectal cancer, bladder cancer, and hepatocellular carcinoma 108-112. SUV39H1 can be methylated at K105 and K123 by SET7/9 21. This modification suppresses the methyltransferase activity of SUV39H1, inhibits H3K9 tri-methylation, and causes relaxation of the heterochromatin condensation 21. MEFs cells with silenced SET7/9 expression were less sensitive to DNase digestion and more resistant to Adr-induced genome instability due to decreased SUV39H1 methylation 21. Furthermore, H1299 cells expressing wild-type SUV39H1 proliferated slower than those expressing SUV39H1-2KR with mutated methylation loci, suggesting that genome instability induced by SUV39H1 methylation restrains cell proliferation and tumor growth 21. The study confirmed the participation of SET7/9 in the modulation of genome stability and provides a new insight into the role of SET7/9 in cancer development 21.

### DNMT1

As one of the three active DNA cytosine methyltransferases, DNMT1 plays a principal role in maintaining the existing methylation marks of CpG sites during DNA replication 113. Deregulation of DNMT1 expression or complete loss of DNMT1 in cancer cells led to hemi-methylation of one fifth of CpG islands in the genome, coupled with G2 cell cycle arrest 114. DNMT1 and SET7/9 are reciprocally regulated, forming a negative feedback loop 40. Both DNMT1 K142 and K1096 can be methylated by SET7/9, but K142 serves as the major target of SET7/9 27,115. SET7/9-mediated DNMT1 K142 methylation caused subsequent proteasome-mediated degradation of DNMT1 and reduced genomic DNA methylation 27,115,116. Meanwhile, DNMT1 represses SET7/9 transcription by methylation of its gene promoter 40. The expression levels of SET7/9 and DNMT1 proteins were inversely correlated in clinical samples of breast cancer 40. What's more, silencing of SET7/9 in breast cancer cells MCF7 reduced cell-cell adhesion and induced EMT transition 40. However, for human cervical cancer cells Hela, though silencing of SET7/9 affected normal cell cycle arrest upon DNA damage, no dramatic phenotypic changes were observed. The current findings imply that the interplay between SET7/9 and DNMT1 may act as a fine-tune mechanism related with epigenetic modulation of gene expression in different cancer types 115.

### HDAC6

Belonging to the histone deacetylases (HDACs) family, HDAC6 is a key regulator of cytoskeleton, stress response, cell motility, and oncogenesis 117. Except for its role in maintenance of de-acetylation balance of histones, HDAC6 also de-acetylates non-histone substrates including α-tubulin and cortactin 118,119. Direct-interaction was detected between SET7 and HDAC6 in CRC cells HCT116 and SW480 120. The interaction did not change the endogenous HDAC6 expression, but inhibited the de-acetylation activity of HDAC6 which further resulted in significantly reduced cell viability and migration on CRC cells 120. SET7/9 overexpression in CRC cells caused HDAC6-dependent inhibition of cell proliferation and wound healing rate 120. In addition, overexpression of SET7/9 reserved the activation effect of HDAC6 on MAPK/ERK signaling 120. This study, together with the study of SET7/9 and Mdm2, has revealed a different way through which SET7/9 can affect protein expression or activity by protein-protein interaction without exerting its lysing methylation function 44,120. However, the inner mechanisms underlying the regulatory effect of SET7/9 on these targets deserve to be further investigated.

### RIOK1

**Figure 8:**
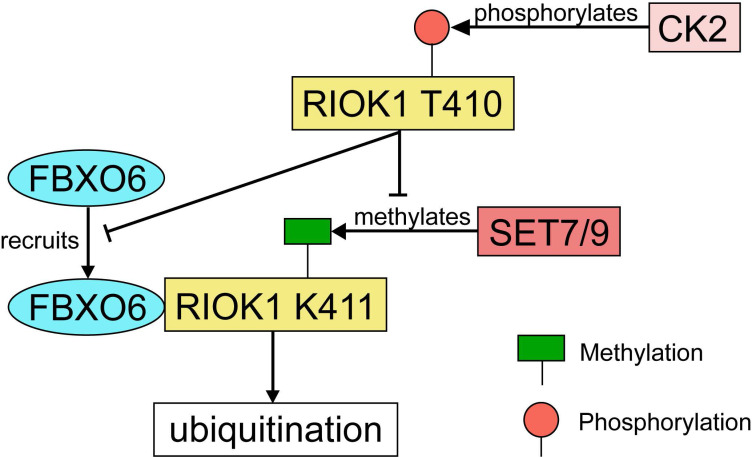
** The regulatory network of RIOK1 by SET7/9, FBXO6, and CK2.** SET7/9 methylates RIOK1 at K411. FBXO6 specifically interacts with K411-methylated RIOK1 through its FBA domain to induce RIOK1 ubiquitination. On the contrary, CK2 phosphorylates RIOK1 T410 and stabilizes the protein by antagonizing K411 methylation and impeding the interaction between FBXO6 and RIOK1.

RIOK1 is a member of the conserved Rio (right open reading frame) family of atypical serine/threonine kinases 121. Although the function of PIOK1 in mammalian cancers has not been well-characterized, RIOK1 upregulation was found to be positively associated with Akt activity in both glioblastoma specimens and cultured cells 122. Meanwhile, a study using isogenic colon-, breast- and lung cancer cell lines has demonstrated that knockdown of RIOK1 strongly impairs cell proliferation and invasiveness 123. In both CRC and gastric cancer tissues, the expression of RIOK1 is significantly upregulated 124. RIOK1 is specifically mono-methylated at the K411 site by SET7/9 and the methylation is reversed by LSD1 124. SET7/9-mediated methylation recruits FBXO6 E3 ligase and contributes to RIOK1 ubiquitination, which finally resulted in reduced tumor growth and metastasis in mice model with CRC 124. On the other hand, RIOK1 T410 is phosphorylated by casein kinase 2 (CK2), which stabilizes RIOK1 by antagonizing K411 methylation and impeding the interaction between FBXO6 and RIOK1 124 (Fig. 8). In support of this, the expression levels of CK2 and LSD1 were inversely correlated with the expression levels of SET7/9 and FBXO6 in human CRC tissues 124.

### Other cancer-related methylation substrates of SET7/9

Beside all the proteins discussed above, SET7/9-catalyzed methylation of some other non-histone proteins with a potential role in cancer development has also been uncovered. For example, TAF10, a subunit of the TFIID transcription factor also known as TAFII30, is efficiently methylated by SET7/9 at K189 in vitro and in vivo 125. Methylation of TAF10 K189 increases its binding capacity to RNA polymerase II and probably stimulates the formation of pre-initiation complex 125. The dynamic interaction between SET7/9 and gene regulatory regions of TAF10 downstream targets enables the activation of specific TAF10-dependent genes, such as *ERF1* and *ERA1* 125. By contrast, methylation of STAT3 at K140 by SET7/9 is a negative regulatory event that blocks activation of many STAT3 target genes, including SOCS3, FGF21, and IRF8/9 126.

Other newly identified substrates of SET7/9 include the p300/CBP-associated factor (PCAF) as well as the farnesoid X receptor (FXR) 127,128. As revealed by *in-vitro* mapping experiments, six lysine residues, K78, K89, K638, K671, K672, and K692 of PCAF as well as K206 of FXR can be methylated by SET7/9 127,128. Undoubtedly, the above methylation events are correlated with gene expression pattern and cellular process. However, whether SET7/9-mediated methylation of these protein targets are directly linked with cellular malignant phenotype and cancer initiation needs to be further investigated.

## SET7/9-mediated histone modification and the biological functions

### SET7/9 catalyzes H3K4me1 in the promoters of *NF-κB* and NF-κB target genes

Despite the fact that SET7/9 functions primarily through the methylation of various non-histone proteins, involvement of SET7/9-mediated histone methylation in epigenetic regulation of gene expression should not been overlooked. SET7/9-catalyzed methylation of histone H3K4, which is known be a pervasive mark of enhancers, has been identified on the promoter regions of many cancer-related genes such as *NF-κB*, *FXR*, and the vascular endothelial growth factor (*VEGF*) 128-131.

A microarray study in TNFα-stimulated human monocytes found that SET7/9 is responsible for the co-activation of one fourth of NF-κB downstream target genes. H3K4 mono-methylation was observed on the promoter regions in several specific inflammatory genes such as *MCP-1*, *IL-8*, and *TNF-α* regulated by NF-κB 128. Activation of these inflammatory genes is closely related with tumor metastasis and angiogenesis 132. It is notable that not all NF-κB downstream targets are regulated by SET7/9. In HEK293 cells and HeLa cells, silencing of SET7/9 suppressed both *IP-10* and *TNF-α* expression, but did not affect *IκBα* expression, suggesting that the regulation of NF-κB signaling by SET7/9 might be promoter-specific 13,20. More recently, H3K4 methylation specifically catalyzed by SET7/9 on NF-κB p65 promoter has also been verified. Epigenetic modification of NF-κB driven by SET7/9 contributes to vascular disorder in patients with type 2 diabetes mellitus 130. To sum up, the current studies supported a role of SET7/9 in both transcriptional and translational regulation of the NF-κB pathway.

### SET7/9 catalyzes H3K4me1 in the promoter of *VEGF*

Vascular endothelial growth factor (VEGF) is an important regulator of tumor angiogenesis frequently up-regulated in cancer 133. By forming a complex with GATA1 that binds to the GATC site in the promoter region of *VEGF*, SET7/9 is able to control GATA1-induced *VEGF* transcription 131. Enrichment of H3K4 mono-methylation was observed at the GATC site in the promoter region of *VEGF* 131. In breast cancer cells, knockdown of SET7/9 not only reduced *VEGF* promoter activity and decreased *VEGF* expression at the mRNA level, but also abolished the ability of GATA1 to activate *VEGF* 131. Furthermore, regulation of cellular behavior, tumor growth, and angiogenesis of breast cancer by GATA-1 is dependent of the function of SET7/9 131. In clinical breast cancer samples, both GATA1 and SET7/9 are overexpressed and their expression levels were significantly correlated with tumor size, grade, and VEGF expression 131.

### SET7/9 regulates H3K4me3 in the promoter of LncRNA DRAIC

Long noncoding RNAs (lncRNAs) are defined as transcripts larger than 200 nt without protein-coding potential. Many lncRNAs bind to chromatin-modifying proteins and recruit their catalytic activity to specific sites in the genome, thereby guide epigenetic regulations in both physiological and pathological conditions 134. Cumulative evidences point to a critical role of lncRNAs in cancer initiation and progression 135-139.

Similar to protein-coding transcripts, transcriptional of lncRNAs is subject to typical histone modification-mediated regulation. DRAIC, also known as LOC145837 and RP11-279F6.1 is the first lncRNA reported to be transcriptionally regulated by SET7/9 140. In human glioma samples, both SET7/9 and lncRNA DRAIC were down-regulated compared with adjacent non-cancerous normal tissues. SET7/9 enhanced H3K4me3 enrichment on the promoter of DRAIC and promoted DRAIC transcription 140. Overexpression of SET7/9 and DRAIC inhibited proliferation, invasion, and migration of U251 cells. The study provided evidences for the involvement of SET7/9 in tri-methylation of H3K4 and suggested a novel mechanism controlling glioma growth and metastasis via the SET7/9-DRAIC axis 140.

### Involvement of SET7/9 in other forms of histone modification

Apart from direct H3K4 methylation, SET7/9 may exert its function as a transcriptional regulator by participating in other forms of histone modification. As mentioned above, SET7/9 selectively activates or represses E2F1 downstream factors *CCNE1* and* p73* 19. Interestingly, although recruitment of SET7/9 to the promoters of these genes was confirmed by ChIP assay, altered SET7/9 expression did not change the level of H3K4 mono-methylation, but increase the level of H3K9 tri-methylation 19. This finding demonstrated the diverse mechanisms through which SET7/9 can regulate gene expression at the transcriptional level.

Besides, the histone methylation targets of SET7/9 are not limited to H3, strong methylation of free H2A and H2B histone proteins, which is comparable with H3 methylation catalyzed by SET7/9 has also been detected by peptide array analysis 24. As a part of histone H3 and H4 carry some modifications before incorporating into chromatin 141, SET7/9-catalyzed methylation of free H2A and H2B may change the dynamic and physiological state of chromatin and affect gene transcription 24. This will be an interesting topic of future study.

### Other genes transcriptionally regulated by SET7/9 via histone modification

Compared with the non-histone targets of SET7/9, gene targets transcriptionally regulated by SET7/9 is less well-understood. However, more and more gene promoters subject to SET7/9-mediated H3K4 methylation or other forms of histone modification are being uncovered. In gastric cancer cells knockdown of SET7/9 inhibited expression of *SREK1IP1*, *PGC*, and *CCDC28B*, accompanied by decreased H3K4me1 level at promoter regions of these genes 142. Accordingly, cell proliferative, migratory, and invasive abilities were significantly increased, and activation of matrix metalloproteinase genes (*MMP1*, *MMP7*, and *MMP9*) was detected 142.

Moreover, chromatin immunoprecipitation-based deep sequencing (ChIP-seq) revealed more than 20,000 SET7/9 specific binding sites in breast cancer cell line MCF-7 143. ChIP assay confirmed the enrichment of SET7/9 to the promoter of *RUNX2*, a transcription factor involved in bone development 143. Depletion of SET7/9 led to decreased *RUNX2* expression and slower tumor growth 143. However, whether the regulatory effect of SET7/9 on *RUNX2* expression results from H3K4 methylation or other types of chromatin modulation merit further investigation.

## Role of SET7/9 in human cancer development

Despite the discovery of various methylation substrates or interactors of SET7/9, most studies of SET7/9 have been limited at the cellular level and the specific role of SET7/9 in different types of human cancer has not been thoroughly examined.

For example, the human osteosarcoma tumor cell line U2OS and human cervical cancer cell line HeLa were frequently used for establishing SET7/9-overexpressing or SET7/9-silencing models to examine the interaction between SET7/9 and its substrates and the cellular biological effects of altered SET7/9 expression. In both cell lines, knockdown of SET7/9 may give rise to a series of malignant phenotype including accelerated cell growth and proliferation, increased cell motility, and reduced cell cycle arrest (Table 1), indicating a potential tumor-suppressor role of SET7/9 in these cells. However, for both human osteosarcoma tumor and cervical cancer, further study on the expression of SET7/9 in clinical tumor samples and the correlation between SET7/9 expression and clinical characteristics remain to be examined to fully confirm the results from *in-vitro* cellular studies.

In human gastric cancer and glioma, both clinical study and cellular analyses support a tumor suppressor role of SET7/9 140,142. SET7/9 enhanced transcription of the *SREK1IP1*, *PGC*, *CCDC28B* genes and the LncRNA DRAIC through H3K4 methylation in the promoter regions and inhibited cell proliferation, migration, and invasion 140,142 (Table 1). Clinically, the expression of SET7/9 was down-regulated in tumor samples of gastric cancer and glioma 140,142. Meanwhile, lower SET7/9 expression is significantly correlated with better survival of patients with gastric cancer 142 (Table 1).

On the contrary, an opposite role of SET7/9 was reported in lung cancer, liver cancer, and intestinal tumor. In liver cancer, SET7/9 expression was significantly higher in clinical tumor sample than in normal tissues 43,144. High SET7/9 expression is positively correlated with tumor metastasis, tumor size, and tumor recurrence 43,144. In consistent with this, silencing of SET7/9 in liver cancer cells resulted in decreased cell proliferation, migration, and invasion 43,144 (Table 1). In lung cancer cells H1299 and A549, many targets of SET7/9 have been identified, including E2F1, Gli3, and Mdm2. Although SET7/9-mediated methylation may either promote or inhibit the degradation of its protein substrates, knockdown of SET7/9 all successfully suppressed the malignant phenotype of lung cancer cells, as revealed by several different studies 19,39,41,44,71 (Table 1). Using an SET7/9-knockdown mice model, a study also showed that SET7/9 controls mice intestinal regeneration and tumorigenesis by regulating Wnt/β-catenin and hippo/YAP signaling 90. It was also found that up-regulation of SET7/9 is associated with increased susceptibility to tumorigenesis 90 (Table). However, similar to the cases of osteosarcoma tumor and cervical cancer, there's a lack of clinical evidences which may further confirm the oncogenic role of SET7/9 in lung cancer and intestinal tumor.

Compared with the cancer types mentioned above, the roles of SET7/9 in breast cancer and colorectal cancer are still controversial. SET7/9 can regulate the cellular biological behavior of breast cancer cells by direct methylation of Estrogen Receptor α or H3K4 methylation in the promoters of *VEGF* and *RUNX2* 12,131,143 (Table 1). Loss of SET7/9 led to decreased cell proliferative and migratory abilities in vitro and inhibited tumor growth and angiogenesis in vivo 12,131,143 (Table 1). Clinically, SET7/9 was found to be an independent poor prognostic factor in breast cancer 131. However, another study detected significantly lower SET7/9 expression in clinical samples of breast cancer and showed that SET7/9 contributes to the epigenetic regulation of epithelial-mesenchymal transition. Knockdown of SET7/9 evoked EMT transition, while overexpression of SET7/9 led to mesenchymal-epithelial transition with upregulated E-cadherin and down-regulated vimentin 40.

For colorectal cancer (CRC), several studies have demonstrated a tumor-suppressing effect of SET7/9 through methylation of RIOK1 and SIRT1 or through direct interaction with HDAC6 50,120,124. Regardless of the different targets identified in these studies, knockdown of SET7/9 all caused increased cell proliferation, migration, and invasion in vitro and enhanced tumor metastasis in vivo 50,120,124. Furthermore, down-regulated SET7/9 expression was detected in clinical tumor samples of CRC, which also indicated worse prognosis 120 (Table 1). However, another study conducted proteomic profiling using serum samples from CRC patients and showed that SET7/9 expression increased from healthy controls to those with colorectal polyps and finally CRC patients. SET7/9 expression was significantly correlated with tumor stage and microsatellite instability, suggesting that SET7/9 may serve as a potential prognostic biomarker for CRC. This study, together with the results from Xie et al. (2011), has supported a tumor-promoting role of SET7/9 in CRC 42,145 (Table 1). The contradictory roles of SET7/9 in breast cancer and colorectal cancer reported by different studies may be due to a limited number of clinical cases or the different genetic background of enrolled patients. Future studies combining large scale clinical analyses, molecular analyses and *in-vitro* and *in-vivo* functional analyses are necessary to validate the function of SET7/9 in these cancer types.

## Potential inhibitors of SET7/9 for pharmacological intervention

Given the critical role of SET7/9 in controlling protein expression, transcription activity, genome stability, cell cycle progression, cell growth and differentiation, SET7/9 has been implicated as a target for cancer therapy. So far, several histone methyltransferase inhibitors have been reported, including DZNep that targets PRC2 146,147 and chaetocin and BIX-01294 that selectively target SUV39 and G9a 148,149.

An X-ray crystal structures of SET7/9 in complex with its potential inhibitor (R)-(3-(3-cyanophenyl)-1-oxo-1-(pyrrolidin-1-yl)propan-2-yl)-1,2,3,4-tetrahydroisoquino-line-6-sulfonamide have been deposited into the Protein Data Bank (Protein database entry 4e47). Two amine analogues of coenzyme S-(5'-adenosyl)-l-methionine (AdoMet), DAAM-3 (DiAzaAdoMet-3) and AAM-1 (AzaAdoMet-1) that bind to the substrate-binding site of SET7/9 and inhibit SET7/9 activity were designed and synthesized 150,151. More recently, another novel potent inhibitor of SET7/9, (R)-PFI-2 was discovered 83. (R)-PFI-2 treatment in murine embryonic fibroblasts (MEFs) caused similar effects with SET7/9 deficiency in the subcellular localization and transcriptional activity of YAP via a substrate-competitive inhibitory mechanism 83. MCF7 cells treated with (R)-PFI-2 demonstrated a dose-dependent increase of nuclear YAP and elevated expression of YAP target genes, *AREG* and *CY61* 83. Thus, (R)-PFI-2 may be used in the modulation of the Hippo pathway by inhibiting SET7/9. However, the effects of these SET7/9 inhibitors on the biological feature of cancer cells haven't been examined.

Apart from all the SET7/9 inhibitors mentioned above, a clinically approved anti-drug, cyproheptadine was found to suppress estrogen-dependent MCF7 cell growth by inhibiting SET7/9 enzymatic activity and abolishing SET7/9-mediated stabilization of ERα 152. The finding suggested a possibility to re-purpose cyproheptadine for breast cancer treatment 152. With more *in-vitro* and *in-vivo* experimental evidences, the SET7/9 inhibitors identified so far may serve as chemical probe tool to interrogate the biological function of SET7/9, or more importantly, as potential chemical agents for pharmacological intervention in clinical treatment of cancer.

## Conclusion and prospect

Methylation-associated modulation of cellular biological processes and signaling pathways have received increased attention in recent years 153,154. As an important methyltransferase, the methylation substrates of SET7/9 vary from histones to non-histone transcription factors, transcriptional co-activators, hormone receptors, DNA cytosine methyltransferases, and other histone methyltransferases (Table 1). Recently, several studies have also demonstrated that SET7/9 can regulate the expression and activities of some proteins simply by protein-protein interaction instead of direct methylation 44,120. By interacting with various substrates and co-regulators, SET7/9 is involved in a complex molecular network underlying epigenetic modulation of gene transcription and regulation of cell cycle, cell motility, differentiation and proliferation, and cell apoptosis. However, due to its broad spectrum of protein targets, SET7/9 may act as either an oncogene or tumor suppressor in different cancer type with different genetic background and cellular contexts. Therefore, the biological and pathological effects of SET7/9 and its chemical inhibitors in each cancer type need to be clarified. Further clinical analysis using tumor samples and functional study using in vivo cancer models may provide a comprehensive view of the role of SET7/9 in cancer initiation and progression, which can help better evaluate the clinical value of SET7/9 as a potential risk predictor or therapy target.

## Figures and Tables

**Table 1:** Functions of SET7/9 and relevant substrates and co-regulators of SET7/9 in different cancer types

Cancer type	Role of SET7/9	SET7/9 expression in clinical sample	Clinical significance	Substrates or interactors	Effects of methylation/ interaction	Biological effects of SET7/9 knockdown
Human breast cancer	Controversial	Overexpression 131	SET7/9 up-regulation is positively correlated with tumor size and grade; Higher SET7/9 expression indicates worse survival 131	VEGF (H3K4) 131	Transcription activation	Decreased cell proliferation, migration, tube formation, tumor growth, and angiogenesis abilities 131
				Estrogen Receptor α (K302) 12	Stabilization of ERα	Attenuated estrogen-driven transcriptional response; Impaired recruitment of ERα to its target genes *PS2* and *PgR* 12
				RUNX2 (H3K4) 143	Transcription activation	Inhibited tumor growth; Inhibited cell migration and invasion 143
		Down-regulation 40	Low SET7/9 expression indicates better overall survival and disease-free survival 40	E2F1 (K185) DNMT1 40	Degradation of E2F1 and DNMT1	Induction of EMT, disruption of cell-cell adhesion and generation of cells with stem cell-like properties 40
Human colorectal cancer	Controversial	Overexpression 145	SET7/9 up-regulation is positively correlated with tumor stage and microsatellite instability 145	E2F1 (K185) 42,145	Stabilization of E2F1	Decreased cell proliferation, migration and invasion 42
				-	-	Decreased cell proliferation; Increased G1/S cell cycle arrest; Increased cell apoptosis 145
		Down-regulation 120, 124	SET7/9 down-regulation is positively correlated with poor prognosis 120, 124	HDAC6 (interaction) 120	Decreased de-acetylation activity of HDAC6	Increased cell proliferation and wound healing rate 120
				RIOK1 (K411) 124	Ubiquitination of RIOK1	Increased cell proliferation, migration, and invasion 124 Enhanced CRC metastasis in vivo
				SIRT1 (K233, K235, K236, K238) 50	Disrupted binding of SIRT1 to p53	Induced p53 deacetylation 50
Human liver cancer	Oncogene	Overexpression 43,144	SET7/9 up-regulation is positively correlated with tumor metastasis, recurrence, large tumor size, and poor tumor differentiation 43,144	E2F1 (K185) 43	Stabilization of E2F1	Decreased cell proliferation, migration and invasion; Decreased expression of E2F1 downstream targets cyclin A2, cyclin E1 and CDK2 43
				ZBTB20 (interaction), CDKN2D (interaction) 144		Increased ZBTB20 and CDKN2D expression; Decreased cell proliferation 144
Human lung cancer	Oncogene	-	-	E2F1(K185) 19,39,41	Degradation of E2F1	Enhanced TP73 expression; Increased cell death; Inhibited cell growth 19,39,41
				Gli3 (K436, K595) 71	Stabilization of Gli3; Increased Gli3-DNA binding capacity.	Suppression of Shh signaling; Inhibited tumor growth and metastasis 71
				Mdm2 (interaction) 44	Increased Mdm2 expression	Defects in DNA repair; Enhanced cell apoptosis; Increased sensitivity to genotoxic stress 44
Mice intestinal tumor	Oncogene	-	SET7/9 up-regulation is positively correlated with tumor susceptibility 90	YAP (interaction) 90	Nuclear accumulation of β-catenin	Decreased expression of Wnt-dependent genes *Lgr5*, *Axin2*, and *Myc*; Diminished Wnt-signaling activation; Fewer intestinal tumors 90
Human gastric cancer	Tumor suppressor	Down-regulation 142	SET7/9 down-regulation is positively correlated with overall survival 142	H3K4 in promoter regions of *SREK1IP1*, *PGC* and *CCDC28B* 142	Enhanced transcription	Increased cell proliferation, migration and invasion; Increased MMPs expression 142
Human glioma	Tumor suppressor	Down-regulation 140	-	H3K4 in the promoter regions of* DRAIC* 140	Enhanced transcription	Increased cell proliferation, migration and invasion 140
Human osteosarcoma tumor	Tumor suppressor	-	-	pRb (K873) 96	Facilitation of pRb-dependent transcriptional repression 96	Increased expression of E2F target genes *E2F1*, *Cdc6*, *DHFR*, *Cdc25A*, and *Cdc2* downstream of pRb; Reduced cell cycle arrest; Induction of senescent cells 96
				pRb (K810) 97, 98	Hypo-phosphorylation of pRb	Increased *DHFR*, *Cdc2*/*6*, *E2F1* expression; Accelerated cell growth 97, 98
				p53 (K372) 18	Stabilization of p53	Inhibited p53 activity; Down-regulation of p21/WAF/CIP and Bax downstream of p53; Defect in cell cycle arrest 18
Human cervical cancer	Tumor Suppressor	-	-	β-catenin (K180) 89	Degradation of β-catenin	Up-regulation of β-catenin and its downstream targets *c-myc* and *cyclin D1*; Increased cell proliferation 89
				HIF-1α (K32) 93	Degradation of HIF-1α	Increased cell motility 93
				DNMT1 (K142, K1096) 115,116	Degradation of DNMT1	Reduced cell cycle arrest 115,116

*Letters and numbers in the brackets indicated the methylation site of SET7/9. K, lysine; H3K4, histone H3 lysine 4; interaction, direct interaction detected between SET7/9 and the protein, but no methylation event was reported.
